# Seasonality of Non-SARS, Non-MERS Coronaviruses and the Impact of Meteorological Factors

**DOI:** 10.3390/pathogens10020187

**Published:** 2021-02-09

**Authors:** Olympia E. Anastasiou, Anika Hüsing, Johannes Korth, Fotis Theodoropoulos, Christian Taube, Karl-Heinz Jöckel, Andreas Stang, Ulf Dittmer

**Affiliations:** 1 Institute for Virology, University Hospital Essen, University of Duisburg-Essen, 45147 Essen, Germany; Ulf.Dittmer@uk-essen.de; 2 Institute of Medical Informatics, Biometry and Epidemiology, University Hospital Essen, University Duisburg-Essen, 45122 Essen, Germany; Anika.Huesing@uk-essen.de (A.H.); k-h.joeckel@uk-essen.de (K.-H.J.); imibe.dir@uk-essen.de (A.S.); 3 Department of Nephrology, University Hospital Essen, University of Duisburg-Essen, 45147 Essen, Germany; johannes.korth@uk-essen.de; 4 Department of Pulmonary Medicine, University Hospital of Essen-Ruhrlandklinik, 45239 Essen, Germany; Fotis.Theodoropoulos@rlk.uk-essen.de (F.T.); christian.taube@rlk.uk-essen.de (C.T.); 5 Centre for Clinical Studies (ZKSE), Institute for Medical Informatics, Biometry and Epidemiology, Medical Faculty, University Duisburg-Essen, 45122 Essen, Germany

**Keywords:** weather, meteorological, coronavirus, immunosuppression, seasonality

## Abstract

Background: Seasonality is a characteristic of some respiratory viruses. The aim of our study was to evaluate the seasonality and the potential effects of different meteorological factors on the detection rate of the non-SARS coronavirus detection by PCR. Methods: We performed a retrospective analysis of 12,763 respiratory tract sample results (288 positive and 12,475 negative) for non-SARS, non-MERS coronaviruses (NL63, 229E, OC43, HKU1). The effect of seven single weather factors on the coronavirus detection rate was fitted in a logistic regression model with and without adjusting for other weather factors. Results: Coronavirus infections followed a seasonal pattern peaking from December to March and plunged from July to September. The seasonal effect was less pronounced in immunosuppressed patients compared to immunocompetent patients. Different automatic variable selection processes agreed on selecting the predictors temperature, relative humidity, cloud cover and precipitation as remaining predictors in the multivariable logistic regression model, including all weather factors, with low ambient temperature, low relative humidity, high cloud cover and high precipitation being linked to increased coronavirus detection rates. Conclusions: Coronavirus infections followed a seasonal pattern, which was more pronounced in immunocompetent patients compared to immunosuppressed patients. Several meteorological factors were associated with the coronavirus detection rate. However, when mutually adjusting for all weather factors, only temperature, relative humidity, precipitation and cloud cover contributed independently to predicting the coronavirus detection rate.

## 1. Introduction

Respiratory tract infections (RTIs) are an important contributor to overall morbidity and mortality, with lower RTI being the fourth most frequent cause of death worldwide [1]. Respiratory viruses are also an important cause of outbreaks and epidemics, including the widespread and in-depth studied influenza virus [2], but also the recently isolated coronavirus, SARS-CoV-2, which continues to have a devastating effect on public health and global economy [3,4]. 

Seasonality is a prominent characteristic of many viral RTIs. In temperate climate regions, viral RTIs reach their peak in winter, and in tropical areas during the rainy season [5,6,7,8]. It has been postulated that the underlying causes of their seasonality are both virus- and host-related. Meteorological factors can influence virus survival, but also modulate human behavior [5,9] and host immune responses [10]. Host-related factors can influence not only the incidence but also the clinical course of an infection. Previous studies have indicated that immunosuppression is a risk factor for an unfavorable outcome in patients with respiratory infections [11,12,13]. Furthermore, there is evidence of prolonged virus shedding in immunocompromised hosts [14], the clinical and epidemiological importance of which remains unclear. 

Interestingly, meteorological factors seem to play a part in the seasonal distribution of some respiratory viral pathogens [6,7]. Preprints of recent studies suggest a potential association between SARS-CoV-2 incidence and climate conditions [15,16,17], but the evidence remains inconclusive and the association is not supported by all available data [18]. In addition, the seasonality and climate dependence of SARS-CoV-2 could not be adequately studied until now, because the ongoing pandemic lasted only a couple of months, and was dramatically influenced by contact distancing measures. The in-depth analysis of the seasonal pattern of non-SARS, non-MERS coronaviruses in conjunction with meteorological factors might therefore provide important insights into the biology of coronaviruses in general. 

The aim of the present study was to evaluate the seasonality and the potential effect of different meteorological factors on the detection rate of the non-SARS, non-MERS coronaviruses. Furthermore, we focused on potential differences in the seasonality of coronaviruses in immunocompetent vs. immunosuppressed hosts. 

## 2. Materials and Methods

We performed a retrospective analysis of 12,763 samples (288 positive and 12,475 negative), including nose/throat swabs, tracheal aspirates and bronchoalveolar lavages, tested in the Institute for Virology of the University Hospital Essen, Germany from June 2013 to December 2019. The samples were tested with the respiratory viral panel (FTD, Siemens, Erlangen, Germany) according to the manufacturer’s instructions, for the detection of non-SARS, non-MERS coronavirus (NL63, 229E, OC43, HKU1). The analysis included all tested samples, meaning that a patient could contribute more than once over time, with the following exception: repeated positive samples from the same individual collected within two weeks of each other were removed. The term “coronavirus” corresponds to non-SARS, non-MERS coronaviruses in our manuscript, unless specifically otherwise noted (e.g., SARS-CoV-2). Nucleic acid extraction was performed using MagNA pure (Roche, Mannheim, Germany). Demographic and clinical data were taken from patient charts. A quarter of our cases (n = 3254, 26.1%) and a third of our positive cases (n = 92, 31.9%) were generated by immunosuppressed individuals (patients with hematological or oncological malignancies under chemotherapy, solid organ transplant recipients, patients after allogeneic human stem cell transplantation).

Meteorological data were obtained for each day of the study period from the weather station of Essen Bredeney, Germany, through the server of “Deutscher Wetterdienst”. The data included daily average temperature, daily average relative humidity, precipitation, daily average wind speed, sunlight hours, daily average cloud cover and daily average atmospheric pressure. We expressed the association between continuous weather variables and the coronavirus detection rates by increments of 5 units on the corresponding scales of the variables if not otherwise specified. The meteorological factors in Essen, Germany for the duration of the study are presented in the supplement (Figure S1). This retrospective study was carried out in accordance with the Declaration of Helsinki and the guidelines of the International Conference for Harmonization for Good Clinical Practice.

### Data Analysis

The virus detection rate was modeled via logistic regression with a seasonal effect and/or weather factors as explanatory variables. Seasonal variation in virus detection rate was fitted for each year separately with a cosinus function, with 1-year frequency length together with a sinus curve, and all variables with a *p*-value below 0.157 (i.e., improving the model quality according to the Akaike information criterion (AIC)) were combined into a single year-specific seasonal score. The formula can be found in the supplement (Supplement Table S1). 

Weather was represented as daily average values locally assessed 10 days prior to the virus measurement to account for incubation time [19,20,21] and time from the first symptoms to coronavirus diagnostis (10 days lag-time). Weather measurements and fitted seasonal effect were compared via Pearson’s correlation analysis.

The effect of single weather factors on the coronavirus detection rate was fitted in a logistic regression model with and without adjusting for other weather factors and seasonality. Variable selection was applied to the weather factors with and without adjustment for season, such as backward, forward, and stepwise selection, targeting statistical model optimization, such as the improvement of AIC. Selected effects were also estimated in patients stratified according to their immune status (immunocompetent vs. immunosuppressed). 

The monthly coronavirus detection rate (as the number of positive samples per tested samples per month) was compared with the corresponding sum of predicted values from the logistic model. Additionally, the coronavirus detection rate was compared with the sum of predicted values within deciles of the predicted values in a calibration plot. As our study does not aim to test or confirm predefined associations, we display the statistical precision of our parameter estimates in graphical presentations of odds ratio estimates with confidence intervals, including a reference line at OR = 1 (as indicating absence of a risk modifying effect). We calculate and report confidence intervals to assess the precision of our estimates, because our goal is estimation and non-significance testing. We wish to avoid publication bias by the preferential reporting of significant results. Instead, we judge the value of our estimates by their precision and validity [22,23]. All statistical analyses were performed using SAS v.9.4 (SAS Institute, Cary, NC USA).

## 3. Results

### 3.1. Coronavirus Infections Followed a Seasonal Pattern

**Figure 1:**
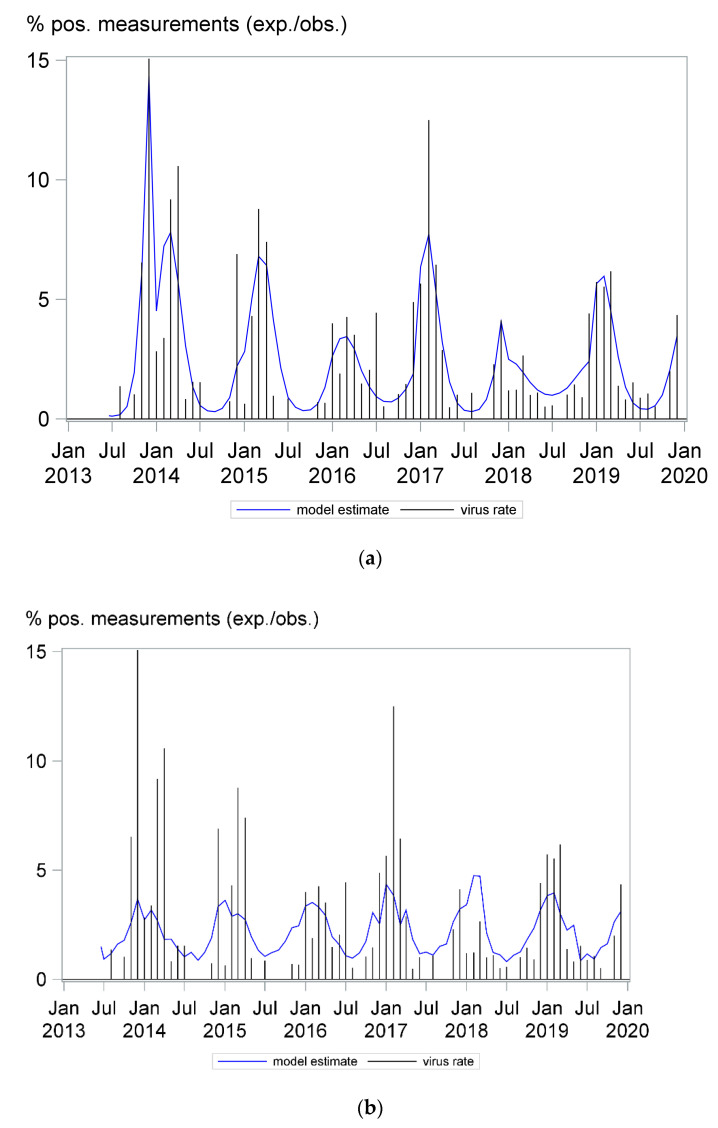
Coronavirus infections followed a seasonal pattern. The needles in the A and B panels show the monthly detection rate. The curves in panels A and B depict the models describing a seasonality effect (**a**) and a combined effect of seven weather factors (daily average ambient temperature, relative humidity, wind speed, cloud cover, atmospheric pressure, precipitation and number of sunlight hours) (**b**) on coronavirus detection rate, respectively. Both models had an adequate fit; however, a better model fit was obtained using seasonality to predict virus detection rates.

Since 2013, we have generated 12,763 test results for the detection of the non-SARS, non-MERS coronaviruses (NL63, 229E, OC43, HKU1) by multiplex PCR technology. Coronavirus infections followed a clear seasonal pattern among our patients. Although coronaviruses were detected throughout the years, detection rates were at their peak from December to March and at their nadir from July to September. In October and November, the number of positive cases was low in most years, usually lower than in May. As shown in Figure 1 the monthly detection rate differed from year to year. A similar seasonal pattern could be observed for the NL63, 229E, and OC43 coronavirus subtypes, as shown in the supplement (Figure S2); an analysis for the HKU1 subtype was not possible due to the low number of detected cases. Furthermore, we divided our patient cohort into three groups according to age, including children and young adults ≤ 20 years old, adults aged from 20 to 60 years old and adults aged more than 60 years old, and fitted the seasonal coronavirus detection rate for each patient group. A clear seasonal pattern could be observed for all three age groups, as seen in Figure S3 in the supplement.

The coronavirus detection rate could be predicted well with a mathematical model including the seasonal effect alone, as well as a model only including seven weather factors (Figure 1a,b, respectively). The model based on seasonal effect was slightly superior in this aspect, fitting the data more accurately, with concordance statistics c = 0.74 for model A and c = 0.68 for model B, and as shown in calibration plots (Figure S4) in the supplement. 

### 3.2. Low Ambient Temperature, Minimum Sunlight Hours per Day, High Relative Humidity, Wind Speed, Cloud Cover and Precipitation Were Each Associated with Higher Detection Rates of Coronavirus

**Figure 2:**
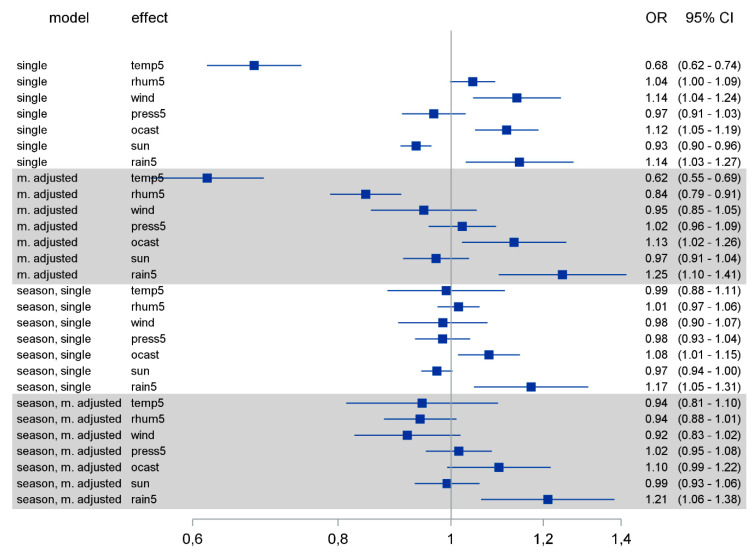
Logistic regression models were calculated for each of the weather factors with and without adjusting for other weather factors and/or seasonality. OR: odds ratio; CI: confidence interval. Temp5: daily average temperature (per 5 °C change); rhum5: daily average relative humidity (per 5% change); wind: daily average wind speed (per 1m/s change); press5: daily average atmospheric pressure (per 5hPa change); ocast: daily average cloud cover; sun: sunlight hours daily; rain5: precipitation daily (per 5 mm change).

We next analyzed the effect of weather factors on coronavirus detection rates. Since coronaviruses including MERS and SARS have an incubation time between 2 and 11 days [19,20,21], we associated the weather factors with infection rates that were diagnosed 10 days later to account not only for the incubation time but also the time passing between the first symptoms and the implementation of diagnostics. The crude effect of single weather factors on the coronavirus detection rate showed associations of lower ambient temperature (Odds Ratio (OR) 0.68, 95% confidence interval (CI) 0.62–0.74), minimum sunlight hours per day (OR 0.93, 95% CI 0.9–0.96), higher relative humidity (OR 1.04, 95% CI 1–1.09) and wind speed (OR 1.14, 95% CI 1.04–1.24), cloud cover (OR 1.12, 95% CI 1.05–1.19) and precipitation (OR 1.14, 95% CI 1.03–1.27) with higher detection rates of coronavirus. Higher atmospheric pressure showed only a weak decreasing effect (Figure 2). We expressed the association between continuous weather variables and the coronavirus detection rates by increments of five units (instead of one unit) on the corresponding scales of the variables daily average temperature, daily average relative humidity, daily average atmospheric pressure and daily precipitation. Since weather factors are correlated with each other to a greater or lesser degree (from correlation (ρ) of 0.01 between temperature and rain to ρ= 0.80 between sunlight and cloud cover; see Table S2 in the supplement), we performed the same analysis for each weather factor after adjusting for all the others. After adjustment, the above-mentioned effects were preserved for ambient temperature (OR 0.62, 95% CI 0.55–0.69), cloud cover (OR 1.13, 95% CI 1.02–1.26) and precipitation (OR 1.25, 95% CI 1.1–1.41). However, in this adjusted analysis, wind speed and hours of sunlight lost impact, low relative humidity (OR 0.84, 95% CI 0.79–0.91) was now associated with higher virus rates, and the atmospheric pressure showed a weak increasing effect.

Most weather factors demonstrate a seasonal pattern (Figure S1). To account for the seasonality effect, we performed the same analysis for each weather factor after adjusting for seasonality, and additionally after adjusting for seasonality and the other weather factors. After adjusting for seasonality, all associations were maintained as before, but largely lost impact. Only the effects of cloud cover and precipitation remained robust. After adjusting for both seasonality and all other weather factors, the point estimates followed the same pattern as in the multiple adjustment above, with cloud cover and precipitation clearly showing increasing effects on virus detection rates (Figure 2). The fact that ambient temperature lost its impact on the viral detection rate in the analysis adjusted for seasonality might be explained by the strong association between temperature and season (Supplement Figure S1 and Table S2).

### 3.3. Temperature, Relative Humidity, Precipitation and Cloud Cover Were Independently Associated with the Coronavirus Detection Rate

**Figure 3:**
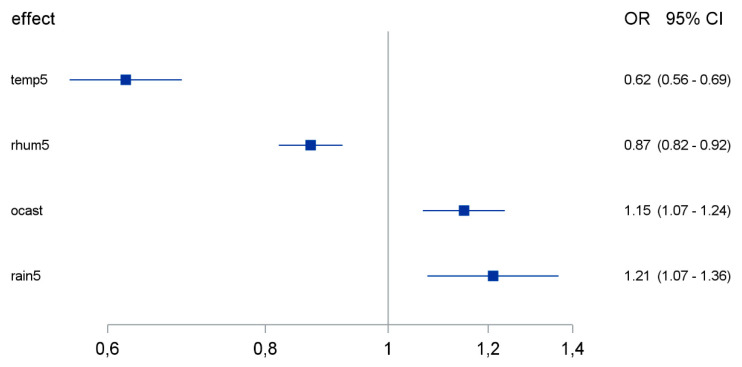
Effect of weather factors on coronavirus detection rate according to a reduced logistic regression model of the combined effect of weather factors. OR: odds ratio; CI: confidence interval. Temp5: daily average temperature (per 5 °C change); rhum5: daily average relative humidity (per 5% change); ocast: daily average cloud cover; rain5: precipitation daily (per 5 mm change).

Next, we aimed to calculate a reduced predictive model for the coronavirus detection rate based on the weather factors. Different automatic variable selection processes agreed to select the predictors of ambient temperature, relative humidity, cloud cover and precipitation as the remaining predictors in the logistic regression model (Figure 3). All above-mentioned factors were independently associated with the coronavirus detection rate, with low ambient temperature, low relative humidity, high cloud cover and high precipitation being linked to increased coronavirus detection rates.

### 3.4. The Seasonality of Coronavirus Infections Was Less Pronounced in Immunosuppressed Patients

**Figure 4:**
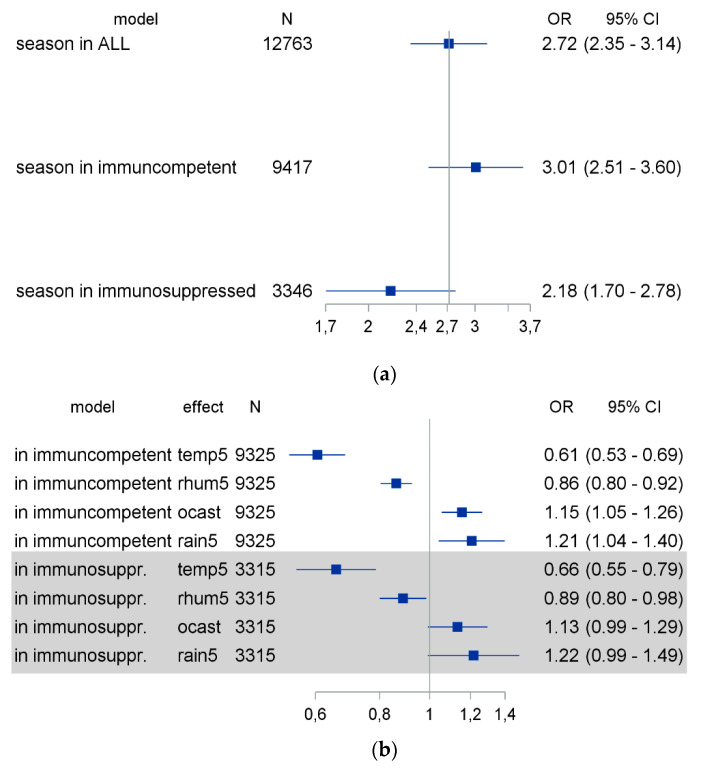
The seasonality and weather influence on coronavirus detection rates was compared between immunosuppressed and immunocompetent patients. Logistic regression models were calculated for the effect of seasonality (**a**) and the effect of selected weather factors (**b**) on the coronavirus detection rate in immunosuppressed and immunocompetent patients. OR: odds ratio; CI: confidence interval; Temp5: daily average temperature (per 5 °C change); rhum5: daily average relative humidity (per 5% change); ocast: daily average cloud cover; rain5: precipitation daily (per 5 mm change); season: grand average year-specific seasonal effect.

A quarter (26.1%) of our patients consisted of immunosuppressed individuals, generating a third (31.9%) of our positive cases. The group of immunosuppressed patients included patients with hematological or oncological malignancies under chemotherapy, solid organ transplant recipients and patients after allogeneic human stem cell transplantation. To evaluate the potential impact of immunosuppression on the seasonality of coronavirus detection, we divided our patients into immunosuppressed and immunocompetent patients, and used logistic regression models to calculate the seasonal effect and the effect of weather factors through a multivariable logistic regression model (Figure 4). As demonstrated in Figure 4, the seasonality effect was less pronounced in cases involving immunosuppressed patients compared to their immunocompetent counterparts, but the selected weather factors had comparable effects in both groups. 

### 3.5. “Off-Season” Coronavirus Detection Was More Frequent in Immunosuppressed Patients

The coronavirus detection rate demonstrated a pronounced seasonal pattern, but even in the time period with the lowest detection rate (summer to fall), we could detect some coronavirus-positive cases. Thus, we aimed to characterize these cases from a clinical point-of-view. 

As shown in Figure 1, the frequency of coronavirus detection was at its nadir from July to September. When analyzing the clinical characteristics of patients with coronavirus infection during its nadir (n = 33), we observed that the majority of positive cases (n = 22, 66%) were immunosuppressed. This included eleven solid organ transplant recipients, six patients after allogeneic bone marrow transplantation and five patients on chemotherapy due to malignant diseases. Information on the travel history of these patients in the last two weeks before coronavirus detection was available for 29 cases, and all but 1 patient had not been abroad. Thus, they must have acquired their coronavirus infection in their local environment, which might have happened weeks before the positive test result. 

Among our patients, data on viral persistence were limited, since follow-up was not consistently performed in most cases. In six cases, we observed viral persistence lasting for more than a month (from 34 to 116 days). Of theses 6 patients, 5 were immunosuppressed.

## 4. Discussion

Coronavirus infections with the viruses NL63, 229E, OC43 and HKU1 followed a seasonal pattern among our patients. Although coronaviruses were in principle detected throughout the year, the detection rate was at its peak from December to March, and at its nadir from July to September. “Off-season” detection of coronavirus was more frequent in immunosuppressed patients. Low ambient temperature, few sunlight hours per day, high relative humidity, wind speed, cloud cover and precipitation were each individually associated with high detection rates of coronaviruses. In a multivariable model including all weather factors, temperature, relative humidity, precipitation and cloud cover were independently associated with the coronavirus detection rate

Seasonality is commonly observed in respiratory infections. Its form and impact depend on the individual pathogen and the climate zone under evaluation [5,6,7,8]. Our study focused on the seasonal non-SARS, non-MERS coronavirus detection rate in Germany, a temperate-climate country. We observed a distinctive seasonal pattern, peaking in winter and plunging in the summer. Our data on seasonality are largely consistent with previous studies evaluating the seasonality of coronavirus [24,25]. However, we show for the first time that data sets on detection rates over many years fit perfectly with a mathematical model of seasonality and weather dependence. 

Seasonality is obviously a characteristic of coronavirus infections. Among our patients, its detection rate was at its peak from December to March and at its nadir from July to September. This pattern has similarities but also differences compared to influenza virus detection. Both viruses peak in the winter, but the influenza virus has a markedly narrower time interval of detection. It is highly prevalent from January to March, but disappears in late spring (usually April), summer and autumn (apart from very few isolated cases), as shown in previous studies [6,7] and in data from the German disease control and prevention agency (Robert Koch Institute) (Supplement Figure S5). Coronavirus, on the other hand, lingers longer during the spring months (until May/June) and appears earlier in the autumn (in November), without completely disappearing over the summer.

A seasonal effect on coronavirus detection rates was present in both immunocompetent and immunosuppressed patents, but it was less pronounced in the immunosuppressed group. “Off-season” detection of coronavirus was more frequent in immunosuppressed patients. Focusing on the “off-season” coronavirus cases, we found that 66% of them were observed in immunosuppressed individuals, although immunosuppressed patients accounted for only 26% of our cases. The difference in the effect of different weather parameters on the coronavirus detection rate in immunosuppressed vs. immunocompetent individuals is minimal. We observed, however, a slightly larger difference in the effect of temperature on the coronavirus detection rate in immunosuppressed vs. immunocompetent hosts, suggesting that temperature indeed may contribute to a greater degree than other weather parameters to coronavirus seasonality, depending on the host’s immune status. It is possible that the observed difference is associated with the fact that temperature demonstrates greater variability throughout the year compared to the other parameters in the model. Since, however, the difference in the above-mentioned effect is very small, caution is warranted in attributing the difference to any particular pathophysiological or behavioral parameter. We have only limited data on the persistence of coronaviruses, but prolonged viral shedding was observed among our patients and it involved mainly immunosuppressed individuals. This is consistent with current knowledge about respiratory virus shedding in humans [14,26], and has been reported in SARS-CoV-2 infection as well [27]. Both phenomena are interesting from a clinical and epidemiological perspective. Coronavirus infections can be detected in the summer months, albeit at very low infection rates. Infections were especially found in immunosuppressed patients, who were reported to be at risk for unfavorable outcomes in respiratory infections [11,12,13]. In addition, prolonged viral shedding may favor the wider distribution of the virus and the emergence of viral variants, as has been reported for other viruses [28]. Immunosuppressed patients may also serve as a reservoir for the virus in the summer. Thus, immunosuppressed hosts may sustain viral replication and spread under otherwise unfavorable external conditions for coronaviruses.

We also evaluated the association of meteorological conditions and coronavirus detection rates. Taken individually, low ambient temperature, minimum sunlight hours per day, high relative humidity, wind speed, cloud cover and precipitation were associated with higher detection rates of coronaviruses 10 days later. Similar weather effects, to a lesser or greater degree, have been described for other respiratory viruses, such as influenza virus and respiratory syncytial virus [7]. In a pediatric population study, the authors looked at the potential effects of some weather factors (temperature, wind velocity, relative humility) on the non-SARS, non-MERS coronavirus detection rate. Each variable was individually evaluated for its potential effect, and indeed a negative correlation between temperature and the coronavirus detection rate, and a positive one for wind velocity and relative humidity, were observed [6]. However, meteorological factors are not independent from one another. Using a multivariable logistic regression analysis and including seven weather factors, we found that only temperature, relative humidity, cloud cover and precipitation were associated with the viral detection rate. Interestingly, relative humidity seemed to have the opposite effect in a multivariable model compared to the univariate one; namely, low relative humidity was associated with higher viral detection rate. The observed effects of temperature and relative humidity are consistent with laboratory data for another coronavirus. SARS-CoV-1 had a better chance of remaining infectious in a low-temperature and low-humidity environment [29]. Our results demonstrate that the coronavirus detection rate is indeed associated with meteorological factors, but also underline the potential pitfalls of analyzing the effects of single factors, without incorporating other interdependent factors in a complex system.

Experimental data on the link between coronavirus transmission and weather factors are largely missing. However, experiments performed with another respiratory virus, e.g., influenza viruses, provide some insight into the possible mechanistic explanation of these associations. We found a clear correlation between coronavirus detection rates and cloud cover or sun light hours. How can this be explained? Solar radiation, which is dependent on cloud cover and sunlight at a given location, has been shown to act in a virucidal way against influenza virus [30]. The virucidal effect of ultraviolet radiation has also been recently demonstrated on SARS-CoV-1 [31]. We also demonstrated a link between temperature and relative humidity and coronavirus detection rates. Temperature can influence virus stability through the inactivation of proteins and nucleic acids [32], but may also influence the host’s defense systems. Cooling and drying of the nasal epithelium inhibits mucociliary clearance and viral phagocytosis [33,34], thus facilitating infections. Low relative humidity can also promote influenza infection and transmission. At lower relative humidity, salts within aerosols tend to crystallize out of the solution, leading to higher virion stability [35], while respiratory tract droplets stay suspended longer and end in the lower respiratory tract more often, thus increasing both the risk of transmission and the risk of an unfavorable infection outcome [32]. 

High precipitation was associated with increased coronavirus detection rates among our patients, which seems counterintuitive when one considers the association of lower relative humidity with higher coronavirus detection rates. Interestingly, the factors of precipitation and relative humidity are not as closely linked (see Supplement, Table S2) as one would expect. A possible explanation is that precipitation does not directly affect viral infectivity or the host’s immune defenses, but mainly influences human behavior, e.g., through increased indoor congregation on a rainy day. Indeed, a similar effect has been described in a study focusing on influenza, where a significant positive association was observed between extreme precipitation and emergency room visits for influenza [36].

Both seasonality and the combined effects of weather factors are reliable predictors of coronavirus detection rates, as shown in the models depicted in Figure 1. This is not surprising, since most weather factors demonstrate a pronounced seasonal pattern (Figure S1). Still, the model based on seasonal effects was slightly superior to the weather model in predicting the viral detection rate. Meteorological factors such as temperature can influence the virus itself, including viability and infectivity, but also effect the host’s immune response [10,37]. Furthermore, they can also modulate human behavior, increasing or decreasing the risk of transmission. Factors potentially contributing to this effect include, but are not limited to, indoor/outdoor activities, indoor heating, crowding, and the impact of melatonin and vitamin D levels on the host’s immune defense [5,9]. The superiority of seasonality as a predictive factor for infection rates suggests that beside the significant correlation with several weather factors, social and cultural norms that modify human behavior also influence viral spread.

A limitation of our study was that we used a convenience sample, namely, patients seeking treatment at our hospital. This leads to the exclusion of infected but asymptomatic individuals, and probably to a marked underestimation of mildly symptomatic individuals, meaning that the true number of coronavirus-infected individuals in the general population at any given time is probably much higher. Furthermore, due to the nature of our study we cannot offer mechanistic explanations for the associations we observed. However, data on the association of weather factors and coronavirus detection rates are very limited, but very important in order to predict the ongoing SARS-CoV-2 pandemic. Our study describes, for the first time, the effect of the interplay of seasonality and several weather factors on the non-SARS, non-MERS coronaviruses’ detection rate, and indicates that some but not all weather factors are independently associated with it, providing valuable insight into the seasonal pattern of coronaviruses in general. This information is relevant for the calculation of projections for disease development according to the time of year and certain weather conditions. An association between meteorological factors and the SARS-CoV-2 detection rate has been suggested [15,16,17], but the evidence remains, to date, inconclusive [18]. The epidemiological situation for SARS-CoV-2 is being further complicated due to drastic lockdown measures all over the world. Our analysis of the seasonal pattern of the non-SARS coronaviruses in conjunction with a potential association with meteorological factors might provide valuable information for the ongoing pandemic. Furthermore, data on the temporal pattern of coronavirus infections in immunosuppressed patients in the literature are limited. Our study allows a first direct comparison of the seasonal pattern and weather effects on the coronavirus detection rate in immunosuppressed versus immunocompetent patients. Our results hint at the relevance of an intact immune system for the epidemiology and seasonality of acute respiratory infections, an aspect that, to the best of our knowledge, has been investigated in a very limited way in the past.

In conclusion, coronavirus infections followed a clear seasonal pattern among our patients, which was more pronounced in immunocompetent patients compared to the immunosuppressed. Several meteorological factors were associated with the coronavirus detection rate. However, after multiple adjustments for all weather factors, only temperature, relative humidity, precipitation and cloud cover remained independently associated with the coronavirus detection rate.

## Data Availability

The data presented in this study can become in part available on request from the corresponding author. The data are not publicly available due to data protection issues.

## Supplementary Materials

The following are available online at https://www.mdpi.com/2076-0817/10/2/187/s1, Figure S1: Weather factors in Essen, Germany. Figure S1A,B depict the average monthly weather factors in Essen, Germany from June 2013 to December 2019. Values are presented as mean ± SD. SD: standard deviation. Table S1: Estimated equation for the outcome season concerning the coronavirus detection rate. Table S2: Correlation between weather factors. Figure S2: Coronavirus infections with different subtypes followed a seasonal pattern. The needles show the monthly detection rate. The curves depict the model describing the seasonality effect. Panel A corresponds to the OC43 subtype, which was detected in 129 samples, panel B to 229E, detected in 92 samples and panel C to the NL63 subtype detected in 59 samples. No analysis was possible for HKU1 due to the low number of positive samples (n = 12). In four samples more than one subtype could be detected (OC43+NL63 in two cases, 229E+OC43, NL63+229E). Figure S3: The coronavirus infection rate followed a seasonal pattern in all three age groups. The needles show the monthly detection rate. The curves depict the model describing the seasonality effect. Panel A corresponds to data from patients aged from 0 to 20 years (n = 89), panel B to data from patients from 20 to 60 years old (n = 111) and panel C to data from patients aged more than 60 years. Figure S4: Panels A and B depict the calibration plot for the prediction of coronavirus detection rate in deciles of predicted values for a model based on a year-specific seasonality effect (A) and a model based on the combined effect of all weather factors (B), respectively. Figure S5: Influenza virus incidence per 100,000 inhabitants in Germany from June 2013 to December 2019. Data are publicly available from the Survstat@RKI 2.0 server. Robert Koch Institut: SurvStat@RKI 2.0, https://survstat.rki.de, Query date: 22.06.2020.

## References

[1] WHO (2018). Global Health Estimates 2016: Disease burden by Cause, Age, Sex, by Country and by Region, 2000–2016.

[2] Dandachi D., Rodriguez-Barradas M.C. (2018). Viral pneumonia: Etiologies and treatment. J. Investig. Med..

[3] Stang A., Standl F., Jöckel K.-H. (2020). Characteristics of COVID-19 pandemic and public health consequences. Herz.

[4] Welfens P.J.J. (2020). Macroeconomic and health care aspects of the coronavirus epidemic: EU, US and global perspectives. Int. Econ. Econ. Policy.

[5] Heikkinen T., Jarvinen A. (2003). The common cold. Lancet.

[6] Du Prel J.B., Puppe W., Grondahl B., Knuf M., Weigl J.A., Schaaff F., Schmitt H.J. (2009). Are meteorological parameters associated with acute respiratory tract infections?. Clin. Infect. Dis..

[7] Price R.H.M., Graham C., Ramalingam S. (2019). Association between viral seasonality and meteorological factors. Sci Rep..

[8] Choe Y.J., Smit M.A., Mermel L.A. (2019). Seasonality of respiratory viruses and bacterial pathogens. Antimicrob. Resist. Infect. Control..

[9] Lofgren E., Fefferman N.H., Naumov Y.N., Gorski J., Naumova E.N. (2007). Influenza seasonality: Underlying causes and modeling theories. J. Virol..

[10] Moriyama M., Ichinohe T. (2019). High ambient temperature dampens adaptive immune responses to influenza A virus infection. Proc. Natl. Acad. Sci. USA.

[11] Branche A.R., Falsey A.R. (2015). Respiratory Syncytial Virus Infection in Older Adults: An Under-Recognized Problem. Drugs Aging.

[12] Shah D.P., Shah P.K., Azzi J.M., Chemaly R.F. (2016). Parainfluenza virus infections in hematopoietic cell transplant recipients and hematologic malignancy patients: A systematic review. Cancer Lett..

[13] Ramsey C.D., Kumar A. (2013). Influenza and Endemic Viral Pneumonia. Crit. Care Clin..

[14] Lehners N., Tabatabai J., Prifert C., Wedde M., Puthenparambil J., Weissbrich B., Biere B., Schweiger B., Egerer G., Schnitzler P. (2016). Long-Term Shedding of Influenza Virus, Parainfluenza Virus, Respiratory Syncytial Virus and Nosocomial Epidemiology in Patients with Hematological Disorders. PLoS ONE.

[15] Merow C., Urban M.C. (2020). Seasonality and uncertainty in COVID-19 growth rates. medRxiv.

[16] Chiyomaru K., Takemoto K. (2020). Global COVID-19 transmission rate is influenced by precipitation seasonality and the speed of climate temperature warming. medRxiv.

[17] Mecenas P., Bastos R., Vallinoto A., Normando D. (2020). Effects of temperature and humidity on the spread of COVID-19: A systematic review. medRxiv.

[18] Bhattacharjee S. (2020). Statistical investigation of relationship between spread of coronavirus disease (COVID-19) and environmental factors based on study of four mostly affected places of China and five mostly affected places of Italy. arXiv.

[19] Lessler J., Reich N.G., Brookmeyer R., Perl T.M., Nelson K.E., Cummings D.A.T. (2009). Incubation periods of acute respiratory viral infections: A systematic review. Lancet Infect. Dis..

[20] Virlogeux V., Fang V.J., Park M., Wu J.T., Cowling B.J. (2016). Comparison of incubation period distribution of human infections with MERS-CoV in South Korea and Saudi Arabia. Sci. Rep..

[21] Backer J.A., Klinkenberg D., Wallinga J. (2020). Incubation period of 2019 novel coronavirus (2019-nCoV) infections among travellers from Wuhan, China, 20–28 January 2020. Eurosurveillance.

[22] Lash T.L. (2007). Heuristic Thinking and Inference From Observational Epidemiology. Epidemiology.

[23] Sterne J.A., Davey Smith G. (2001). Sifting the evidence-what’s wrong with significance tests?. BMJ.

[24] Lau S.K.P., Woo P.C.Y., Yip C.C.Y., Tse H., Tsoi H.-W., Cheng V.C.C., Lee P., Tang B.S.F., Cheung C.H.Y., Lee R.A. (2006). Coronavirus HKU1 and other coronavirus infections in Hong Kong. J. Clin. Microbiol..

[25] Nickbakhsh S., Ho A., Marques D.F.P., McMenamin J., Gunson R.N., Murcia P.R. (2020). Epidemiology of Seasonal Coronaviruses: Establishing the Context for the Emergence of Coronavirus Disease 2019. J. Infect. Dis..

[26] Van der Vries E., Stittelaar K.J., van Amerongen G., Veldhuis Kroeze E.J., de Waal L., Fraaij P.L., Meesters R.J., Luider T.M., van der Nagel B., Koch B. (2013). Prolonged influenza virus shedding and emergence of antiviral resistance in immunocompromised patients and ferrets. PLoS Pathog..

[27] Man Z., Jing Z., Huibo S., Bin L., Fanjun Z. (2020). Viral shedding prolongation in a kidney transplant patient with COVID-19 pneumonia. Am. J. Transplant..

[28] Vega E., Donaldson E., Huynh J., Barclay L., Lopman B., Baric R., Chen L.F., Vinjé J. (2014). RNA Populations in Immunocompromised Patients as Reservoirs for Novel Norovirus Variants. J. Virol..

[29] Chan K.H., Peiris J.S.M., Lam S.Y., Poon L.L.M., Yuen K.Y., Seto W.H. (2011). The Effects of Temperature and Relative Humidity on the Viability of the SARS Coronavirus. Adv. Virol..

[30] Sagripanti J.-L., Lytle C.D. (2007). Inactivation of Influenza Virus by Solar Radiation. Photochem. Photobiol..

[31] Darnell M.E.R., Subbarao K., Feinstone S.M., Taylor D.R. (2004). Inactivation of the coronavirus that induces severe acute respiratory syndrome, SARS-CoV. J. Virol. Methods.

[32] Marr L.C., Tang J.W., Van Mullekom J., Lakdawala S.S. (2019). Mechanistic insights into the effect of humidity on airborne influenza virus survival, transmission and incidence. J. R. Soc. Interface.

[33] Eccles R. (2002). An Explanation for the Seasonality of Acute Upper Respiratory Tract Viral Infections. Acta Oto-Laryngol..

[34] Salah B., Dinh Xuan A.T., Fouilladieu J.L., Lockhart A., Regnard J. (1988). Nasal mucociliary transport in healthy subjects is slower when breathing dry air. Eur. Respir. J..

[35] Yang W., Elankumaran S., Marr L.C. (2012). Relationship between humidity and influenza A viability in droplets and implications for influenza’s seasonality. PLoS ONE.

[36] Smith G.S., Messier K.P., Crooks J.L., Wade T.J., Lin C.J., Hilborn E.D. (2017). Extreme precipitation and emergency room visits for influenza in Massachusetts: A case-crossover analysis. Environ. Health.

[37] Hayashi O., Kikuchi M. (1989). Time relationship between ambient temperature change and antigen stimulation on immune responses of mice. Int. J. Biometeorol..

