# Supplementary material for: Seasonality of Non-SARS, Non-MERS Coronaviruses and the Impact of Meteorological Factors
Source: Pathogens. 2021 Feb 9;10(2):187. doi: 10.3390/pathogens10020187 (PMC7916144; doi:10.3390/pathogens10020187)
Supplement: Supplementary file 1 [file pathogens-10-00187-s001.pdf]

Supplement

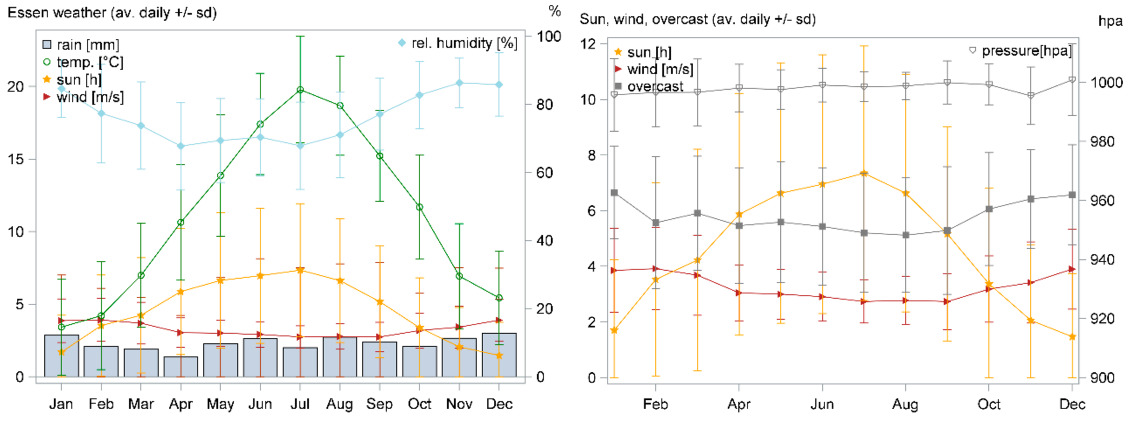

Figure S1: Weather factors in Essen, Germany. Figure S1A and B depict the average monthly weather factors in Essen, Germany from June 2013 to December 2019. Values are presented as mean±SD. SD: standard deviation.

Table S1: Parameter estimates for seasonal calendar effect (per calendar week) on the outcome of Corona virus detection rate in a logistic model.

Year	Estimated equation for the outcome season
2013	1.5789 * cos((6.283185308/52)*(week - 9 - 0.5)) - 2.1464 * sin((6.283185308/52)*(week - 9 - 0.5))
2014	1.7072 * cos((6.283185308/52)*(week - 9 - 0.5))
2015	1.4852 * cos((6.283185308/52)*(week - 9 - 0.5)) + 0.4968 * sin((6.283185308/52)*(week - 9 - 0.5))
2016	0.8244 * cos((6.283185308/52)*(week - 9 - 0.5))
2017	1.5616 * cos((6.283185308/52)*(week - 9 - 0.5)) - 0.6142* sin((6.283185308/52)*(week - 9 - 0.5))
2018	0.2456 * cos((6.283185308/52)*(week - 9 - 0.5)) - 0.4102* sin((6.283185308/52)*(week - 9 - 0.5))
2019	1.1880 * cos((6.283185308/52)*(week - 9 - 0.5)) - 0.7662* sin((6.283185308/52)*(week - 9 - 0.5))

The binary outcome of corona-infection yes/no among a varying number of patients was fitted as a logistic regression model on parameters indicating seasonally changing effects of calendar-date. To keep the model as simple as possible with the aim of robustness and generalizability we selected only those parameters, which improved the model according to the Akaike-information criterion (AIC) [i.e. with a p-value below 0.157]. Seasonal variation was modelled as a cosinus function with a period length of 1 year, starting from the time of peak incidence, an additional sinus function allowed for attenuation in shape. Yearly variation in the strength of these seasonal parameters clearly improved the model fit. The selected parameters were combined into a single year-specific seasonal score as a fixed comparison factor for further analyses of weather effects.

Table S2: Correlation between weather factors.

ρ 95% CI	Seasonal effect	Tempera-ture (°C)	Wind speed (m/s)	Atmospheric pressure (hpa)	Relative Humidity, %	Precipitation, mm	Sunlight hours	Cloud cover (okta)
Seasonal effect	-	-0.62 (-0.65- -0.59)	0.25 (0.2- 0.29)	-0.07 (-0.12--0.03)	0.10 (0.06- 0.15)	-0.03 (-0.07-0.02)	-0.21 (-0.26- -0.17)	0.1 (0.06- 0.15)
Temperature (°C)	-0.62 (-0.65- -0.59)	-	-0.22 (-0.27- -0.18)	0.02 (-0.02-0.07)	-0.52 (-0.55- -0.48)	-0.01 (-0.05-0.04)	0.50 (0.47- 0.54)	-0.25 (-0.29- -0.2)
Wind speed (m/s)	0.25 (0.20 - 0.29)	-0.22 (-0.27- -0.18)	-	-0.37 (-0.41--0.33)	0.04 (-0.00- 0.09)	0.25 (0.21-0.29)	-0.23 (-0.27- -0.18)	0.18 (0.14- 0.23)
Atmospheric pressure (hpa)	-0.07 (-0.12 - -0.03)	0.02 (-0.02- 0.07)	-0.37 (-0.41- -0.33)	-	-0.14 (-0.18- -0.09)	-0.32 (-0.36 - -0.28)	0.25 (0.21- 0.30)	-0.30 (-0.34- -0.26)
Relative Humidity, %	0.10 (0.06 - 0.15)	-0.52 (-0.55- -0.48)	0.04 (-0.00- 0.09)	-0.14 (-0.18--0.09)	-	0.31 (0.27-0.35)	-0.78 (-0.79- -0.76)	0.59 (0.56- 0.62)
Precipitation, mm	-0.03 (-0.07 - 0.02)	-0.01 (-0.05- 0.04)	0.25 (0.21- 0.29)	-0.32 (-0.36 - -0.28)	0.31 (0.27- 0.35)	-	-0.30 (-0.34- -0.26)	0.29 (0.24- 0.33)
Sunlight hours	-0.21 (-0.26 - -0.17)	0.50 (0.47- 0.54)	-0.23 (-0.27- -0.18)	0.25 (0.21-0.30)	-0.78 (-0.79- -0.76)	-0.30 (-0.34- -0.26)	-	-0.80 (-0.81- -0.78)
Cloud cover (okta)	0.1 (0.06 - 0.15)	-0.25 (-0.29- -0.2)	0.18 (0.14- 0.23)	-0.30 (-0.34- -0.26)	0.59 (0.56- 0.62)	0.29 (0.24- 0.33)	-0.80 (-0.81- -0.78)	-

Seasonal effect refers to the average year specific seasonal effect (see Figure 1). All other variables refer to daily average values except for precipitation and sunlight hours. We performed Pearson´s correlation; the values within the cells depict the correlation coefficients (ρ) and 95 % confidence interval (CI).

A

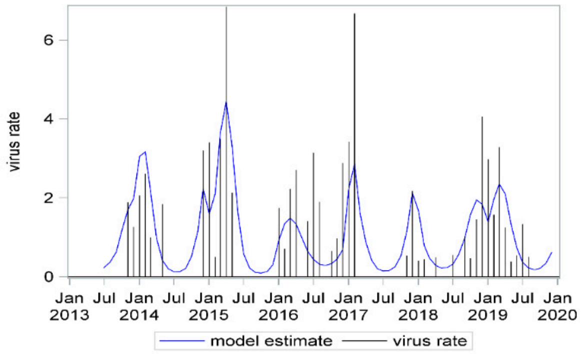

B

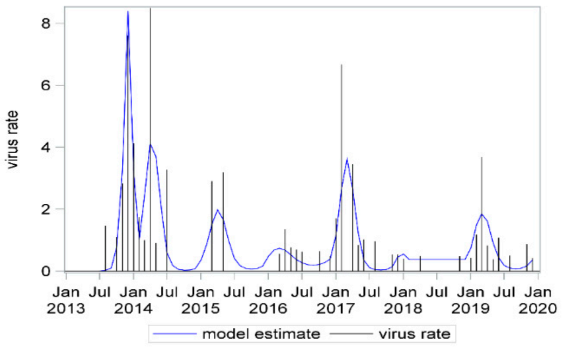

C

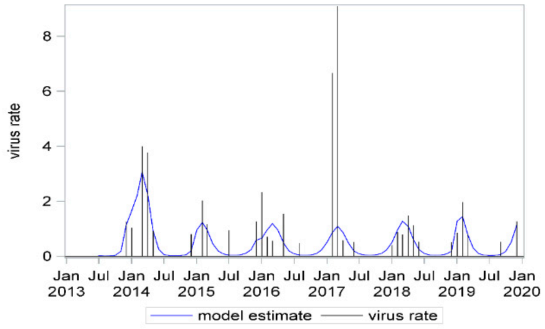

Figure S2. The Corona virus infection rate of different subtypes followed a seasonal pattern. The needles show the monthly detection rate. The curves depict the model describing the seasonality effect. Panel A corresponds to the OC43 subtype, which was detected in 129 samples, panel B to 229E, detected in 92 samples and panel C to the NL63 subtype detected in 59 samples. No analysis was possible for HKU1 due to the low number of positive samples (n=12). In four samples, more than one subtype could be detected (OC43+NL63 in two cases, 229E+OC43, NL63+229E).

A

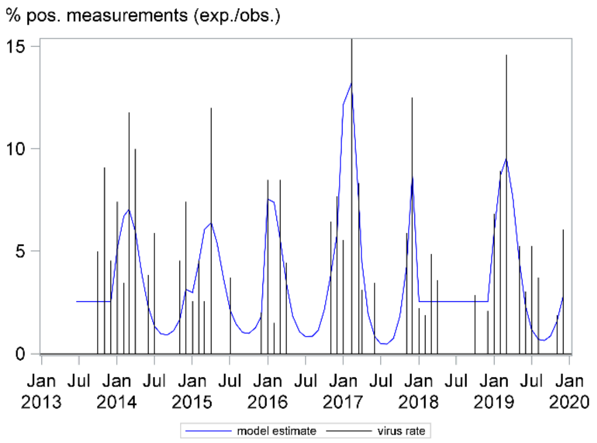

B

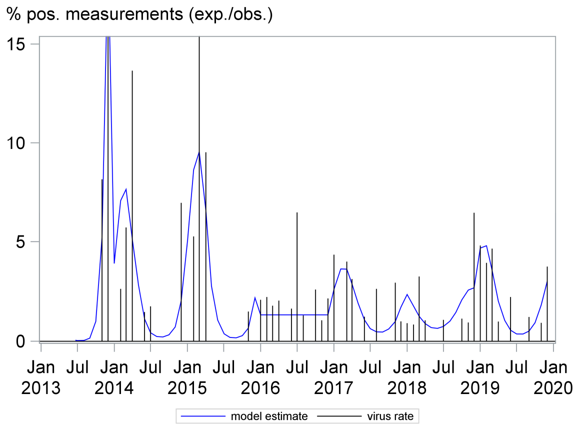

C

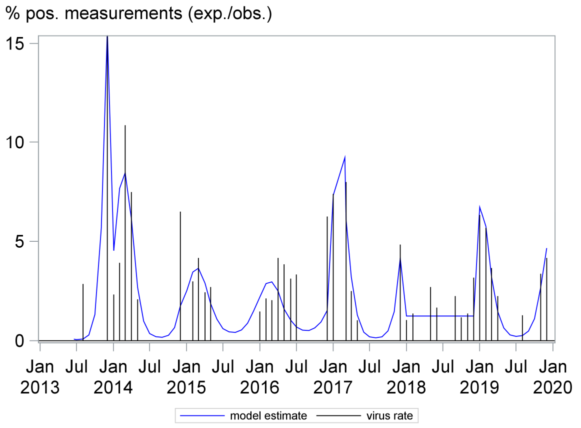

Figure S3: The Corona virus infection rate followed a seasonal pattern in all three age groups. The needles show the monthly detection rate. The curves depict the model describing the seasonality effect. Panel A corresponds to data from patients aged from 0 to 20 years (n=89), panel B to data from patients from 20 to 60 years old (n=111) and panel C to data from patients aged more than 60 years.

A **B**

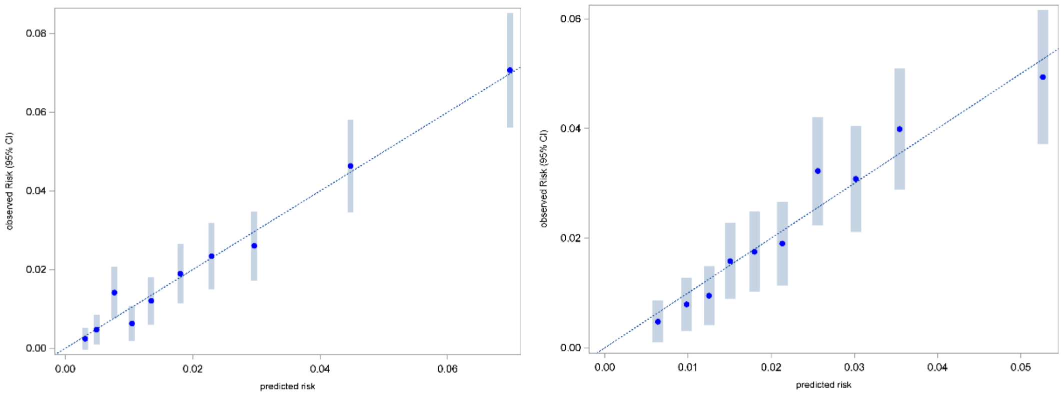

Figure S4: Panels A and B depict the calibration plot for the prediction of Corona virus detection rate in deciles of predicted values for a model based on a year-specific seasonality effect (A) and a model based on the combined effect of all weather factors (B) respectively.

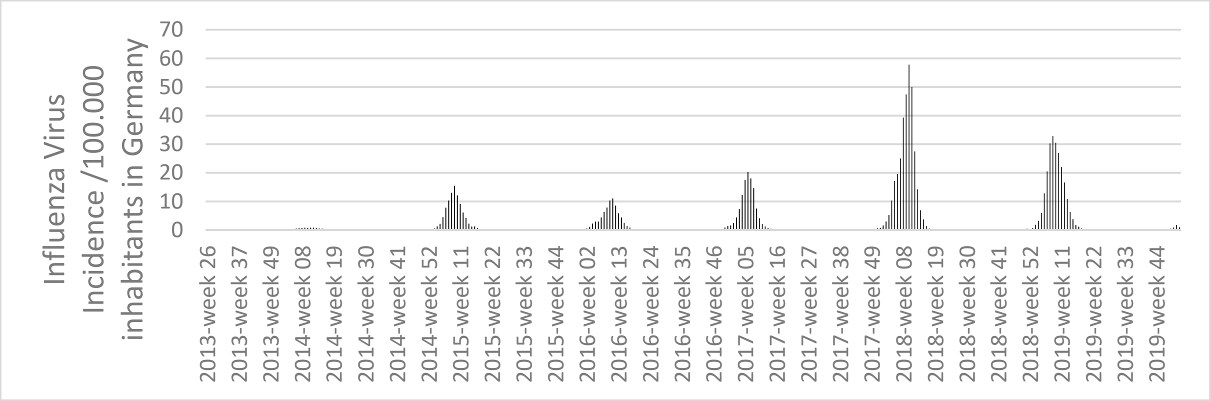

Figure S5: Influenza virus incidence per 100.000 inhabitants in Germany from June 2013 to December 2019. Data are publicly available from the Survstat@RKI 2.0 server. Robert Koch-Institut: SurvStat@RKI 2.0, https://survstat.rki.de, Query date: 22.06.2020.
